# Impact of forward and backward walking on gait parameters across parkinson’s disease stages and severity: a prospective observational study

**DOI:** 10.1186/s12883-025-04321-2

**Published:** 2025-09-09

**Authors:** Tracy Milane, Nicolas Vuillerme, Pascal Petit, Elke Warmerdam, Robbin Romijnders, Edoardo Bianchini, Walter Maetzler, Clint Hansen

**Affiliations:** 1 Department of Neurology, University Hospital Schleswig-Holstein, Kiel, Germany; 2 Univ. Grenoble Alpes, AGEIS, Grenoble, 38000 France; 3 Institut Universitaire de France, Paris, France; 4 Department of Neuroscience, Mental Health and Sensory Organs (NESMOS), Sapienza University of Rome, Rome, Italy

**Keywords:** Parkinson’s disease, H&Y, Severity, Gait, Backward walking, Step length

## Abstract

**Background:**

Parkinson’s disease (PD) is characterized by motor symptoms altering gait domains such as slow walking speed, reduced step and stride length, and increased double support time. Gait disturbances occur in the early, mild to moderate, and advanced stages of the disease in both backward walking (BW) and forward walking (FW), but are more pronounced in BW. At this point, however, no information is available about BW performance and disease stages specified using the Hoehn and Yahr (H&Y) scale. The objectives of this study were to examine the link between clinical scores and gait parameters in PD, and to assess gait parameters in both FW and BW among PD patients in early disease stages (H&Y: 1–2) and advanced disease stages (H&Y: 3–4), as well as among PD patients with mild and moderate disease severity as per the Movement Disorder Society-Unified Parkinson’s Disease Rating Scale Part III (MDS-UPDRS III).

**Methods:**

Spatiotemporal gait parameters were analyzed during FW and BW over a 5-meter walkway at a comfortable speed using 3D motion capture. Correlations and regressions between clinical scores and gait parameters were examined. Wilcoxon Mann-Whitney rank sum tests were used to compare PD patients in early and advanced disease stages and assess differences in gait parameters for both FW and BW conditions.

**Results:**

The study included a total of 25 PD patients (aged 65 ± 9 years), among whom 10 were in the H&Y stages 1–2 and 15 in stages 3–4. All participants were evaluated with the MDS-UPDRS III, with 17 having a total score ≤ 32 (mild impairment and disability) and 8 having a total score > 32 (moderate impairment and disability). During BW, PD patients with H&Y stages 1–2 had significantly (*p* < 0.05) longer step lengths, stride lengths, and a higher walk ratio compared to those with H&Y stage 3–4. Regardless of the walking condition, no difference was found between PD patients with a MDS-UPDRS III total score ≤ 32 and patients with a MDS-UPDRS III total score > 32.

**Discussion:**

The study demonstrates that individuals with PD in H&Y stages 3–4 exhibit compromised FW and BW abilities in comparison to those in stages 1–2. Notably, the disparities are more prominent in the realm of backward walking. These findings substantiate the existence of distinct gait patterns between the early and advanced stages of the disease, with the variations being particularly accentuated in the context of backward walking.

**Conclusions:**

Taken together, our results suggest that backward walking may hold greater clinical utility in assessing and managing PD patients.

**Trial registration:**

The research procedure was approved by the ethical committee of the Medical Faculty of Kiel University (D438/18). The study is registered in the German Clinical Trials Register on 20,200,904 (DRKS00022998).

**Supplementary Information:**

The online version contains supplementary material available at 10.1186/s12883-025-04321-2.

## Introduction

Parkinson’s disease (PD) is a progressive neurodegenerative disorder characterized primarily by motor symptoms, including bradykinesia, rigidity, and resting tremor [5]. These fundamental motor impairments can lead to modifications in gait patterns [5]. Gait alterations observed in PD encompass reduced walking speed [27], shorter stride/step lengths [5, 10], prolonged double support time [5], increased cadence [27], and are notably prevalent in the early stages of PD [18]. These disturbances, although partially responsive to dopaminergic therapy [9], are less treatable than other motor manifestations and, as the disease advances, they intensify in severity and become less responsive to conventional treatment methods [9]. This elevates the risk of falls [41] and diminishes the quality of life for patients [34]. Modifications in gait parameters are closely linked to disease progression, with prior studies predominantly focusing on PD patients in the advanced disease stages rather than in the early stages [21]. Furthermore, various types of gait disturbances can be distinguished across the early, mild to moderate, and advanced stages of the disease [10].

While forward walking (FW) is considered a fundamental task in human locomotion [36], daily life entails various directional movements, including side-stepping and backward walking (BW). Notably, BW is a more challenging and demanding form of ambulation due to increased postural instability and the absence of visual cues [24]. PD patients have exhibited gait disturbances in both the FW and BW [4]. However, previous research has predominantly concentrated on FW, given its prevalence as the most common type of walking [21]. Yet, BW plays a significant role in activities of daily living, such as stepping back from a sink or navigating tight spaces like closets [3].

Recent investigations [13, 35, 36] have reported that the differences in gait parameters between PD patients and healthy adults are more pronounced in BW than in FW. Indeed, the inability to perform backward steps effectively can increase the risk of falls [11]. Our own recent findings synthesized from two systematic reviews also support this notion, as we observed that in PD patients, BW parameters could be linked to cognitive functions [6]. Additionally, we found that PD patients experiencing freezing of gait (FOG) exhibited poorer BW performance compared to those without FOG [26].

Therefore, the assessment of PD patients in BW holds clinical significance, as BW is a vital aspect of mobility [11]. Consequently, gait analysis in PD should extend beyond FW to encompass other functional walking tasks, particularly BW, given the valuable insights it can provide [12].

Only a limited number of studies have delved into the correlation between gait characteristics and Hoehn & Yahr (H&Y) stages among PD patients during FW [2, 8, 15, 41, 45]. The H&Y scale stands as the most widely employed tool for assessing the stage of PD, utilizing a scale of 1 to 5 to gauge the clinical disability levels of PD patients [16]. This scale categorizes stages into three groups: mild (stages 1–2), moderate (stage 3), and severe (stages 4–5) [25]. Conversely, while previous research has specifically addressed BW [13, 14, 29, 30, 35–37], to the best of our knowledge, no investigation has explored the relationship between BW and the clinical scores of PD patients. Investigating the relationship between BW and disease progression could add relevant information to the global status of PwPD. Indeed, BW is a walking pattern more cognitively demanding and in PD [6], cognitive disturbances usually increase in severity and prevalence along disease progression [1] as well as motor disturbances and axial symptoms (Poewe et al. 2017) This could lead to increased risk of falls and injuries. Therefore it could be hypothesized that assessment of BW over time could provide valuable information on the mobility of PwPD beyond the mere movement assessment.

Therefore, the objectives of this study were twofold: (1) to examine the link between clinical scores and gait parameters in PD, and (2) to assess gait parameters in both FW and BW among PD patients in early disease stages (H&Y: 1–2) and advanced disease stages (H&Y: 3–4), as well as among PD patients with mild and moderate disease severity as per MDS-Unified Parkinson's Disease Rating Scale Part III (MDS-UPDRS III).

## Methods

The aim of this retrospective observational study was to examine the link between clinical scores and gait parameters in PD, and to assess gait parameters in both FW and BW among PD patients in early disease stages (H&Y: 1–2) and advanced disease stages (H&Y: 3–4). This study is reported following the STROBE guidelines [https://www.strobe-statement.org/].

This study employed a retrospective observational design, using a subset of data previously recorded from a larger cohort. The data were originally collected for the validation of IMU or 3D motion capture based algorithms since this validation should be performed per disease since different mobility limitations or symptoms can influence the performance of an algorithm in different ways. The collected dataset contains data from both healthy participant and patients with neurological diseases (Parkinson’s disease, stroke, multiple sclerosis, chronic low back pain). In total 167 participants were measured with IMUs and an optical motion capture (reference) system. Participants performed multiple standardized mobility assessments and non-standardized activities of daily living. For this study, the subset was selected based on Parkinson’s disease diagnosis. No new data were collected as part of this analysis.

### Ethics approval and reference number

The original study received ethical approval from the Ethics Committee of Kiel University (D438/18) and was conducted according to the guidelines of the Declaration of Helsinki and approved by the and all participants provided written informed consent before participation. The study is registered in the German Clinical Trials Register on 2020-09-04 (DRKS00022998). This secondary analysis of previously collected data did not require additional ethics approval, as per guidelines from Ethics Committee of Kiel University.

### Study setting and participant recruitment

The original data were collected between 09/2021 and 01/2022 from healthy subjects who were recruited via flyers that were placed in public facilities. The neurological patients were recruited from either the outpatient clinic or the University Hospital Schleswig-Holstein neurology ward, Campus Kiel, Germany. A total of 167 subjects were measured but the sample size was inherently limited to the available data, and it was not possible to collect additional participants. This secondary analysis was conducted using data recorded within this timeframe. Data analysis for the current study was performed between January 2024 and August 2024.

### Inclusion and exclusion criteria

The study’s participant selection criteria encompassed individuals aged 18 years and older who could ambulate independently without requiring walking aids. Exclusion criteria comprised a Montreal Cognitive Assessment (MoCA) score below 15. For this study, a subset of 34 participants was selected based on their Parkinson’s disease diagnosis. To categorize the participants, they were first divided into two groups based on their H&Y stage within the Parkinson’s disease (PD) spectrum: those in stages 1–2 and those in stages 3–4. Individuals classified as H&Y stage 5 (“Wheelchair bound or bedridden unless aided”) were excluded from the research, as they did not meet the prerequisite of independent ambulation without walking aids.

Furthermore, another grouping criterion was the MDS-UPDRS III score. Participants with an MDS-UPDRS III score greater than 32 were categorized separately from those with scores less than or equal to 32, utilizing the defined cutoff scores established to characterize mild to moderate disease severity, as outlined by Martínez-Martín P et al. (2015) [25].

### Sample size calculation

The sample size for this study was predetermined based on prior research and the current analysis is a secondary analysis of the previously published data set [24, 25].

#### Data collection

A twelve-camera optical motion capture system (Qualisys AB, Göteborg, Sweden) was used to record full-body movements at 200 Hz. A total of 47 reflective markers (19 mm) were adhered to the body for the walking assessments. During the static calibration trials, eight additional reflective markers (19 mm) were placed on the body (elbows, knees and ankles) to be able to estimate joint positions The over ground walking was performed on a walkway with a width of 1 m. The start and end of the 5 m during which steady state gait is recorded will be marked by cones with reflective markers (30 mm) on top of them. A minimum of three markers were placed on the body segments: head, sternum, upper arms, forearms, hands, lower back, thighs, shanks, and feet see Table 1. For a detailed explanation of the technical setup and the data collection we refer the reader to [43, 44].

**Table 1:** Provides a detailed description about the marker placement during the experimental gait sessions in the clinical gait laboratory

Location	Exact description of the location
front of the head	Located approximately over the temple
back of the head	Placed on the back of the head, roughly in a horizontal plane of the front head markers
sternum	Placement mid of sternum, just caudal of SC joints
sternum	Caudal and to the left of sternum
sternum	Caudal and to the right of sternum
shoulder	Placed on the acromio-clavicular joint
upper arm	Placed between elbow and shoulder (left side closer to plane of the front head markers)
elbow	Placed on lateral epicondyle approximating elbow joint axis
humerus	Placed on top of humerus epicondylus medialis
forearm	Placed between wrist and elbow (left side closer to wrist, right side closer to elbow)
wrist, radial side	Placed on the radial styloid
wrist, ulnar side	Placed on the ulnar styloid
dorsal side of hand	Dorsum of the hand just proximal of the head of the second metacarpal
anterior superior iliac spine	Placed directly on top of anterior superior iliac spine
posterior superior iliac spine	Placed directly on top of posterior superior iliac spine
thigh	Marker cluster on lateral side of thigh, contains four markers
knee (medial)	Placed on the medial epicondyle of the knee
knee (lateral)	Placed on the lateral epicondyle of the knee
lateralis	
shank	Marker cluster on lateral side of shank, contains 4 markers
ankle (medial)	Placed on the malleolus medialis
ankle (lateral)	Placed on the malleolus lateralis
heel	Placed on the calcaneous
toe	Placed on the shoe around the 2nd middle phalanx

#### Gait protocol

The gait protocol involved the assessment of walking performance under different conditions. The assessments were conducted on a 5-meter walkway in a clinical gait laboratory in their respective ON-phase. Prior to each assessment, participants were instructed to start walking two steps before the start of the walkway and to continue walking two steps after the end of the walkway. Participants were allowed to familiarize themselves with the task prior to the actual measurement. The participants were always closely monitored by an examiner who remained within arm’s reach to ensure safety in case of any trips or stumbles.

**Fig. 1:**
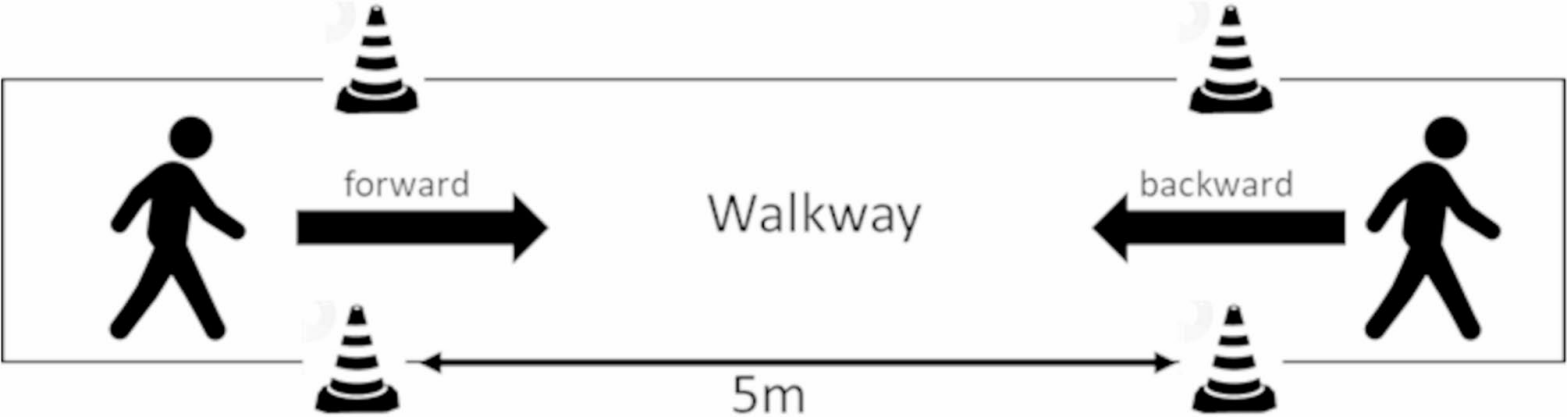
Shows the experimental setup of the 5 m walking path on the walkway in the clinical gait laboratory for the forward and the backward walking trials

The following two walking conditions were assessed at convenient speed: (1) Participants were instructed to walk forward at comfortable speed and (2) participants were instructed to walk backward along the same 5-meter walkway (Fig. 1).

**Fig. 2:**
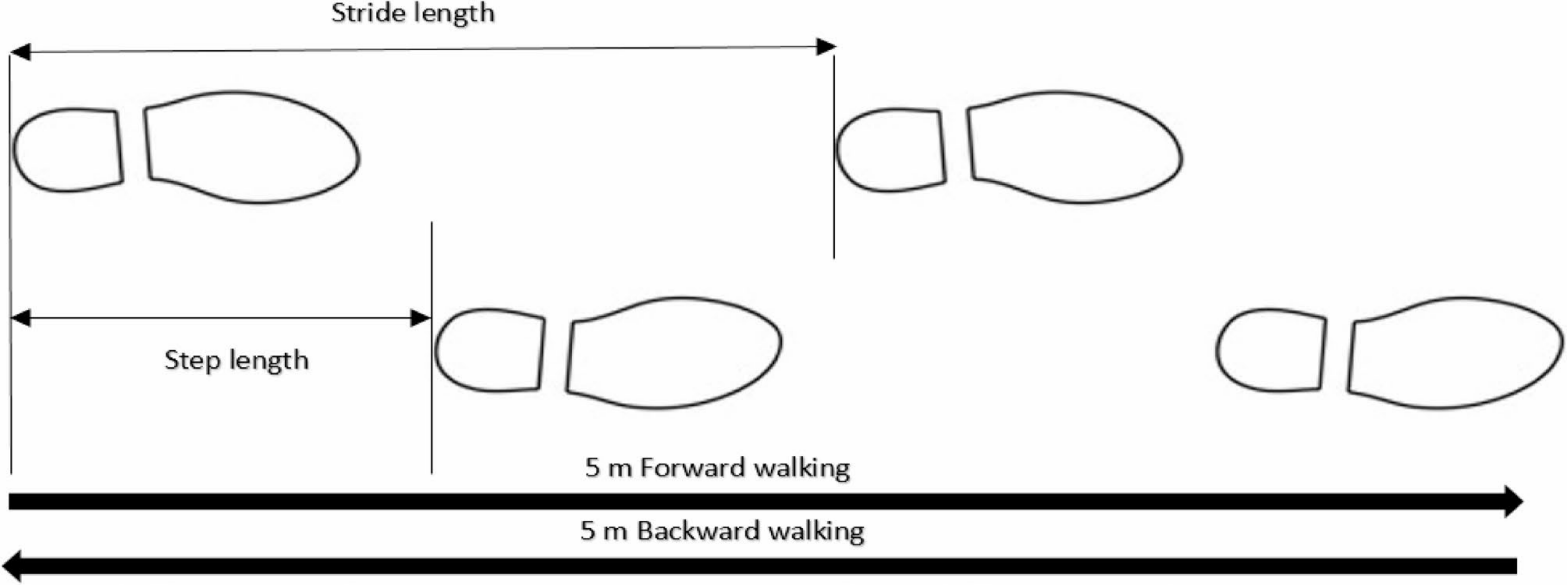
Step length and stride length description

For each walking condition, the extracted parameters included step and stride velocity, step and stride length, step frequency, swing duration, walk ratio (i.e. step length divided by cadence), and walking direction effect (WDE) (Fig. 2). WDE was calculated to assess the influence of the walking direction (backward vs. forward walking) for each gait parameter. WDE was calculated as a relative measure of change (WDE), between the backwards gait parameters and the forward gait parameters (e.g., step length).

WDE has the same unit as the gait parameter that is used in the calculation, inspired by [38]. A negative value of WDE for a given gait parameter (e.g., step length) is associated to better performance in the forward walking condition than in the backward walking condition, while a positive WDE value is associated to worse performance in the forward walking condition than in the backward walking condition. The WDE was calculated as a unitless percentage (WDE%) to enable comparison across gait parameters. This was determined by subtracting forward gait parameters from backward gait parameters, dividing the result by the forward gait parameters, and multiplying by 100. In addition to the gait parameters, clinical scores were also recorded for each participant. The H&Y score, which assesses the stage of PD, and the total score of MDS-UPDRS III, evaluating motor symptoms severity, were obtained.

### Data analysis

Descriptive statistics were calculated for the extracted gait parameters and clinical scores and expressed as arithmetic means and standard deviations (mean ± sd) expect for H&Y and MDS-UPDRS III which are reported as median and (Q1-Q3). The variables sex, handedness and FOG were expressed as total number of participants (n) and percentages (%).

### Statistical methods

Pearson’s correlation (between continuous variables) and polyserial correlation (between categorical and quantitative variables) analyses were performed to examine the associations between gait parameters and clinical scores. A coefficient < 0.1 indicates a negligible correlation, 0.1–0.39 a weak correlation, 0.40–0.69 a moderate correlation, 0.70–0.89 a strong correlation, and > 0.9 a very strong correlation [33].

A linear regression analysis was conducted to further investigate the relationships between each gait parameter and clinical score. Initially, a simple linear regression analysis was undertaken, as follows: clinical PD score (H&Y or UPDRS III) ~ gait parameter_i_ in walking condition_j_ (j: FW or BW). Then, a multivariate linear regression analysis was performed by considering each gait parameter in both walking conditions, as follows: clinical PD score (H&Y or UPDRS III) ~ gait parameter_i_ in FW + gait parameter_i_ in BW. Finally, for each gait parameter, an interaction test was conducted to statistically evaluate walking condition differences by adding an interaction term in the multivariate linear regression model (FW*BW).

Wilcoxon Mann-Whitney rank sum tests were employed to compare PD patients in early and advanced disease stages and assess differences in gait parameters for both FW and BW conditions. The Wilcoxon effect size (ES) and its 95% confidence interval were computed to quantify the difference between the two groups beyond p-value interpretation. To that end, Vargha and Delaney’s A statistic (VDA) was used. VDA is a standardized quantification of the difference between two groups. VDA reports the probability that a value from one group will be greater than a value from the other group. A value of 0.50 indicates that the two groups are stochastically equal. A value of 1 indicates that the first group shows complete stochastic dominance over the other group. A value of 0 indicates the complete stochastic dominance of the second group. Vargha and Delaney (2000) [40] suggested an ES of 0.45–0.55 as a negligible effect, 0.56–0.63 (or 0.35–0.44) as a small effect, 0.64–0.70 (or 0.30–0.34) as a medium effect, and > 0.70 (or < 0.30) as a large effect. The type I error rate was set at α = 0.05.

No a priori sample size estimation was performed, as the dataset from [43, 44] was utilized for the analysis. Data analysis was performed using R software 4.3.1^®^ (R Core Team, Vienna, Austria) for Windows 10^©^.

## Results

Originally 34 PD patients, were initially included, not all were able to complete the backward walking task hence 25 the study included a sample of 25 PD patients, consisting of 17 males and 8 females (Table 2). The mean age of the participants was 65 ± 9 years, with a mean disease duration of 9 ± 6 years among whom 10 were in the H&Y stages 1–2 and 15 in stages 3–4. All participants were evaluated with the MDS-UPDRS III, with 17 having a total score ≤ 32 (mild impairment and disability) and 8 having a total score > 32 (moderate impairment and disability).

**Table 2:** Demographics of the participants, overall and for each disease stage and disease severity group

	All participants (*n* = 25)	H&Y 1–2 (*n* = 10)	H&Y 3–4 (*n* = 15)	MDS-UPDRS III score ≤ 32 (*n* = 17)	MDS-UPDRS III score > 32 (*n* = 8)
Sex, male (n, %)	17 (65%)	6 (60%)	11 (73%)	11 (65%)	6 (75%)
Age (years) (mean ± sd)	65 ± 9	62.1 ± 7.49	67.3 ± 9.93	66 ± 10	64 ± 8
Disease duration (years) (mean ± sd)	9 ± 6	9.50 ± 5.46	8.33 ± 7.06	9 ± 6	9 ± 8
Height (cm) (mean ± sd)	175 ± 9	173 ± 7.54	177 ± 9.75	176 ± 9	175 ± 10
Weight (kg) (mean ± sd)	83 ± 18	79.9 ± 15.4	86.0 ± 19.5	84 ± 17	83 ± 23
BMI (kg/m²) (mean ± sd)	27 ± 5	26.7 ± 4.78	27.1 ± 4.67	27 ± 4	27 ± 6
Foot length (cm) (mean ± sd)	29 ± 2	28.6 ± 1.52	29.7 ± 2.05	29 ± 2	29 ± 2
Right handed (n, %)	16 (61%)	5 (50%)	9 (67%)	9 (53%)	6 (75%)
Left handed (n, %)	2 (8%)	2 (20%)	0	2 (12%)	0
FOG (n, %)	5 (19%)	1 (10%)	3 (20%)	2 (12%)	2 (25%)
H&Y score (median [Q1-Q3])	3 [2–3]	1.5 [1.0–2]	3 [3–3]	2 [1.5-3]	3 [3–4]
MDS-UPDRS III total score (median [Q1-Q3])	25 [12–40]	11.5 [10.5–23.0]	36.0 [24.5–48.5]	20 [11–25]	48.5 [43-65.8]

*BMI* Body Mass Index, *FOG* Freezing of gait, *H&Y* Hoehn and Yahr scale, *MDS-UPDRS III* Motor part of the MDS-Unified Parkinson's Disease Rating Scale part 3. All variables are reported as mean (standard deviation) or number (percentage) expect for H&Y and MDS-UPDRS III which are reported as median and (Q1-Q3)

The H&Y stage was assessable for 25 participants, comprising 10 (40%) in stages 1–2 and 15 (60%) in stages 3–4. Evaluation with the MDS-UPDRS III involved 25 participants, of whom 17 (68%) scored ≤ 32 (mild impairment/disability) and 8 (32%) scored > 32 (moderate impairment/disability).

### Correlations between FW, BW and clinical scores

#### Correlation analysis regarding the MDS-UPDRS III total score

For FW, there was a negative correlation between the MDS-UPDRS III total score and step velocity (*r*=−0.46; *p* = 0.017), stride velocity (*r*=−0.50; *p* = 0.012), step length (*r*=−0.47; *p* = 0.014), and stride length (*r*=−0.46; *p* = 0.019) (Table S1). No significant correlation was found for any gait parameters in BW.

### Correlation analysis regarding the H&Y score

For FW, a notable correlation was observed between the stride length and the H&Y score (R2 = 0.09, *p* = 0.047), indicating a potential positive association. Conversely, for BW, no parameter demonstrated a significant correlation with the H&Y score (Table S2).

### Linear regression

#### Simple linear regression

The results of the simple linear regression analysis aligned with those of the correlation analysis, confirming the abovementioned associations. During FW, the stride length was associated with the H&Y score (R2 = 0.17, *p* = 0.047), while the step velocity (R2 = 0.24, *p* = 0.02), step length (R2 = 0.25, *p* = 0.01), stride velocity (R2 = 0.26, *p* = 0.01), and stride length (R2 = 0.23, *p* = 0.02) were associated with the MDS-UPDRS III total score. By contrast, no gait parameters were associated with the H&Y and MDS-UPDRS III total scores during BW (Table S3, sheet entitled “Simple linear regression”).

#### Multiple linear regression

When considering the H&Y or MDS-UPDRS III total scores, regardless of the gait parameter considered, no multiple regression linear analysis that included both FW and BW (gait parameter = FW + BW) yielded significant results (Table S3, sheet entitled “Multiple linear regression), which indicated that considering both FW and BW failed to explain the variability of H&Y or MDS-UPDRS III total scores.

### **Comparison between MDS-UPDRS III score** ≤ **32 vs. MDS-UPDRS III score > 32**

Table 3 presents the results of the Wilcoxon Mann-Whitney rank sum tests used to examine differences in gait parameters in FW and BW between the two groups of PD severity. No significant difference in median values was found between the two groups for FW, and BW. In FW, there was a trend (*p* < 0.10) with large effect size towards a higher stride length (*p* = 0.09) and walk ratio in MDS-UPDRS III score ≤ 32 (*p* = 0.09) compared with MDS-UPDRS III score > 32.

**Table 3:** Differences between the two groups of MDS-UPDRS III total score in forward and backward walking

Gait parameters	Forward walking					Backward walking				
	MDS-UPDRS III total score ≤ 32 (*n* = 17)	MDS-UPDRS III total score > 32 (*n* = 8)	W	*p*	ES [95% CI]	MDS-UPDRS III total score ≤ 32 (*n* = 17)	MDS-UPDRS III total score > 32 (*n* = 8)	W	*p*	ES [95% CI]
Step frequency [steps per minutes]	97.5 ± 11.4	100 ± 12.1	59	0.98	0.49 [0.26; 0.73]	106 ± 13.6	98.2 ± 19	67	0.33	0.64 [0.38; 0.84]
Step velocity [m/s]	1.11 ± 0.23	0.94 ± 0.22	83	0.15	0.69 [0.45; 0.86]	0.58 ± 0.26	0.53 ± 0.27	59	0.68	0.56 [0.31; 0.79]
Step length [m]	0.57 ± 0.10	0.49 ± 0.10	85	0.12	0.71 [0.47; 0.87]	0.30 ± 0.15	0.28 ± 0.11	60	0.63	0.57 [0.32; 0.79]
Step duration [s]	0.74 ± 0.27	0.67 ± 0.24	58	0.93	0.48 [0.25; 0.72]	0.56 ± 0.08	0.72 ± 0.31	35	0.24	0.33 [0.15; 0.59]
Stride velocity [m/s]	1.12 ± 0.23	0.94 ± 0.20	85	0.12	0.71 [0.47; 0.87]	0.58 ± 0.27	0.54 ± 0.30	55	0.89	0.52 [0.28; 0.76]
Stride length [m]	1.25 ± 0.22	1.08 ± 0.21	87	0.09	0.73 [0.49; 0.88]	0.64 ± 0.31	0.62 ± 0.26	59	0.68	0.56 [0.31; 0.79]
Stride duration [s]	1.13 ± 0.09	1.17 ± 0.09	42	0.27	0.35 [0.16; 0.60]	1.11 ± 0.17	1.23 ± 0.23	35	0.24	0.33 [0.15; 0.59]
Swing velocity [m/s]	2.59 ± 0.85	2.38 ± 0.38	37	0.70	0.57 [0.28; 0.82]	0.10 ± 0.07	0.10 ± 0.08	51	0.91	0.52 [0.27; 0.76]
Swing length [m]	0.97 ± 0.28	0.93 ± 0.16	38	0.63	0.59 [0.29; 0.83]	0.08 ± 0.05	0.07 ± 0.04	51	0.91	0.52 [0.27; 0.76]
Swing duration [s]	0.40 ± 0.10	0.39 ± 0.03	24	0.44	0.37 [0.15; 0.67]	0.77 ± 0.12	0.86 ± 0.20	37	0.40	0.38 [0.17; 0.64]
Stance duration [s]	0.68 ± 0.05	0.70 ± 0.04	25	0.50	0.39 [0.16; 0.68]	0.36 ± 0.05	0.38 ± 0.05	35	0.32	0.36 [0.16; 0.62]
Walk ratio	5.9e-3 ± 1.4e-3	5.0e-3 ± 1.1e-3	87	0.087	0.73 [0.49; 0.88]	2.9e-3 ± 1.5e-3	2.8e-3 ± 9.5e-4	52	1.00	0.50 [0.25; 0.74]

*MDS-UPDRS III* Motor part of the MDS-Unified Parkinson's Disease Rating Scale part 3. All variables are reported as mean (standard deviation) or number (percentage) expect for H&Y and MDS-UPDRS III which are reported as median and (Q1-Q3). W: Wilcoxon test statistics, p: v-value, ES: the Wilcoxon effect size

### Comparison between H&Y stages 1–2 vs. H&Y stages 3–4

Table 4 presents the results of the Wilcoxon Mann-Whitney rank sum tests used to examine differences in gait parameters in FW and BW between H&Y stage 1–2 and H&Y stage 3–4 groups.

**Table 4:** Differences between the two groups of H&Y in forward and backward walking

Gait parameters	Forward walking					Backward walking				
	H&Y (1–2) (*n* = 10)	H&Y (3–4) (*n* = 15)	W	*p*	VDA [95% CI]	H&Y (1–2) (*n* = 10)	H&Y (3–4) (*n* = 15)	W	*p*	VDA [95% CI]
Step frequency [steps per minutes]	95 ± 10.7	98.1 ± 12.7	36	0.65	0.43 [0.20; 0.69]	101 ± 13.9	105 ± 16.8	33	0.66	0.43 [0.20; 0.70]
Step velocity [m/s]	1.11 ± 0.16	0.99 ± 0.23	56	0.26	0.67 [0.40; 0.86]	0.68 ± 0.19	0.48 ± 0.25	56	0.13	0.73 [0.46; 0.89]
Step length [m]	0.58 ± 0.07	0.51 ± 0.10	60	0.14	0.71 [0.45; 0.88]	**0.37 ± 0.10**	**0.24 ± 0.12**	**62**	0.035*	**0.81 [0.57; 0.93]**
Step duration [s]	0.82 ± 0.29	0.71 ± 0.27	51	0.48	0.61 [0.34; 0.82]	0.60 ± 0.08	0.57 ± 0.11	51	0.29	0.66 [0.39; 0.86]
Stride velocity [m/s]	1.12 ± 0.16	1.00 ± 0.23	59	0.17	0.70 [0.44; 0.88]	0.68 ± 0.19	0.49 ± 0.27	56	0.13	0.73 [0.46; 0.89]
Stride length [m]	1.28 ± 0.19	1.12 ± 0.19	62	0.10	0.74 [0.48; 0.90]	**0.80 ± 0.20**	**0.53 ± 0.29**	**61**	0.044*	**0.79 [0.55; 0.92]**
Stride duration [s]	1.14 ± 0.08	1.15 ± 0.11	36	0.65	0.43 [0.20; 0.69]	1.20 ± 0.15	1.13 ± 0.23	51	0.29	0.66 [0.39; 0.86]
Swing velocity [m/s]	2.77 ± 0.56	2.32 ± 0.84	30	0.36	0.67 [0.35; 0.88]	0.12 ± 0.05	0.09 ± 0.07	46	0.32	0.66 [0.38; 0.86]
Swing duration [s]	0.37 ± 0.04	0.42 ± 0.11	16	0.44	0.36 [0.13; 0.67]	0.81 ± 0.12	0.81 ± 0.19	38	0.81	0.54 [0.27; 0.79]
Stance duration [s]	0.71 ± 0.03	0.67 ± 0.05	33	0.19	0.73 [0.43; 0.91]	0.39 ± 0.04	0.35 ± 0.05	51	0.13	0.73 [0.46; 0.89]
Walk ratio	6.2e-3 ± 1.3e-3	5.3e-3 ± 1.4e-3	61	0.12	0.73 [0.47; 0.89]	**3.8e-3** ± **1.1e-3**	**2.4e-3** ± **1.2e-3**	**61**	0.044*	**0.79 [0.55; 0.92]**

* Significant difference between groups within walking condition; All variables are reported as mean (standard deviation) VDA: Vargha and Delaney’s A statistic as is a standardized effect size quantification of the difference between two groups. H&Y: Hoehn and Yahr scale.

#### Backward walking characteristics

With a large effect size, PD patients with H&Y stages 1–2 had significantly longer step length (0.38 ± 0.13 vs. 0.24 ± 0.12 [m], *p* = 0.01), stride length (0.80 ± 0.26 vs. 0.52 ± 0.27 [m], *p* = 0.02) and a higher walk ratio (3.6e-3 ± 1.3e-3 vs. 2.4e-3 ± 1.2e-3, *p* = 0.04), stride velocity (0.70 ± 0.25 vs. 0.47 ± 0.25 [m/s], *p* = 0.04), and step velocity (0.70 ± 0.24 vs. 0.46 ± 0.43 [m/s], *p* = 0.04) compared to those with H&Y stage 3–4 (Table 4).

#### Forward walking characteristics

For FW, encouraging trends emerged, indicating that PD patients in H&Y stages 1–2 exhibited potentially higher step length (*p* = 0.096), stride length (*p* = 0.053), and stride velocity (*p* = 0.07) compared to those in stages 3–4 (Table 4).

#### Summary of key findings

To summarize our main results suggest that in BW, PD patients with H&Y stages 3–4 had more gait impairment compared with those with H&Y stages 1–2 e.g. highlighted by significantly longer step length (0.38 ± 0.13 vs. 0.24 ± 0.12 [m], *p* = 0.01), stride length (0.80 ± 0.26 vs. 0.52 ± 0.27 [m], *p* = 0.02) and a higher walk ratio (3.6e-3 ± 1.3e-3 vs. 2.4e-3 ± 1.2e-3, *p* = 0.04), stride velocity (0.70 ± 0.25 vs. 0.47 ± 0.25 [m/s], *p* = 0.04), and step velocity (0.70 ± 0.24 vs. 0.46 ± 0.43 [m/s], *p* = 0.04) compared to those with H&Y stage 3–4 (Table 4).

## Discussion

The objectives of this study were twofold: (1) to examine the link between clinical scores and gait parameters in PD, and (2) to assess gait parameters in both FW and BW among PD patients in early disease stages and advanced disease stages, as well as among PD patients with mild and moderate disease severity as per the MDS-UPDRS III. During BW, PD patients with H&Y stages 1–2 had significantly (*p* < 0.05) longer step lengths, stride lengths, and a higher walk ratio compared to those with H&Y stage 3–4. Regardless of the walking condition, no difference was found between PD patients with a MDS-UPDRS III total score ≤ 32 and patients with a MDS-UPDRS III total score > 32.

PD patients often exhibit gait impairments in spatiotemporal parameters, both in BW and FW, with more pronounced deficits observed in BW compared to healthy individuals [12]. Our results indicate that PD patients classified in H&Y stages 3–4 have shorter stride length, step length, and lower walk ratio during BW compared to their counterparts in stages 1–2. This result aligns with the findings reported by Hackney and Earhart, who investigated PD patients with mild to moderate severity (H&Y: 1–3) in the ON medication state. These authors observed that, during BW versus FW, participants exhibited slower walking speeds, shorter strides, a reduced swing percentage, increased stance and double support percentages, a wider base of support, and lower functional ambulation performance [13, 14]. Our study corroborates these findings and introduces a novel observation of significant differences between patients in earlier and more advanced stages of PD. Notably, in BW, PD patients categorized within H&Y stages 3–4 displayed shorter stride lengths compared to patients in stages 1–2, with no statistically significant differences observed in step frequency between these two groups.

This observation implies that a reduction in gait speed might be another distinguishing characteristic of individuals in H&Y stages 3–4. In response to the progressive disease-related shortening of step and stride length [5, 10], PD patients have been reported to increase their cadence to a greater extent than healthy elderly [22]. Slower walking speeds may offer increased stability and reduced fall risk, providing more time to react to potential tripping hazards [7]. Prior research [19] suggests that individuals with concerns about falling often adopt a more cautious gait characterized by slower speed, shorter step length, an increased base of support, and longer double support. Considering the significance of stride length, measuring a patient’s stride length in FW could hold substantial value in determining walking limitations [28]. The longer step length, stride length, higher walk ratio, and increased stride and step velocities observed in the more advanced PD group (H&Y 3–4) during BW, in contrast to the less advanced group (H&Y 1–2), likely reflect compensatory strategies employed by individuals with greater disease severity when walking backward. Backward walking demands a different set of motor control processes compared to forward walking, requiring increased reliance on proprioceptive feedback and potentially engaging different neural pathways [24]. In PD, a reduction in proprioception has been reported [20] and particularly ankle proprioception was linked to functional mobility and disease stage [42]. In addition, gait is linked with cognition, particularly executive, frontal functions [46]. In PD patients, a lower executive functions performance has been reported [1] and the impact of this alteration is reflected by the marked negative impact of motor-cognitive dual-task on gait parameters in PD patients [31].

In more advanced PD, where forward gait is often characterized by shorter steps, shuffling, and reduced velocity, individuals might instinctively adopt a different pattern when walking backward to maintain stability or initiate movement. This might explain the reported increase in step and stride length which is supported by [39] who showed that BW gait requires a higher level of attention and poses a heavier cognitive load. Therefore gait alterations could be more pronounced in BW than in FW in patients with PD, as discussed in a recent systematic review [6].

Moreover, Hackney & Earhart [13], proposed that the neural correlates implemented for BW, including cortical and brainstem areas, could be impacted earlier in people with PD patients. The higher walk ratio observed in the more advanced PD group during BW, a measure that typically increases with gait impairment, suggests greater instability or altered control mechanisms specific to backward ambulation. This indicates that the relationship between cadence and step length is affected in BW and may be more sensitive to the progression of motor symptoms. These observations could the association between motor symptom severity and BW parameters, while no such association was apparent for FW.

However, it is important to acknowledge that our lack of observed associations between BW parameters and other clinical variables could be attributed to several factors. The limited sample size might have restricted our statistical power to detect subtle but significant relationships. Furthermore, the limited variability in the UPDRS scores may have restricted the ability to observe correlations between BW performance and clinical severity. These factors collectively might have contributed to the absence of detectable associations. Incorporating a larger sample size, such as different subtypes (tremor dominant and postural Instability/gait difficulty groups), and different stages (H&Y 1, 2, 3, 4, or even prodromal PD) may be more helpful in elaborating the gait characteristics of patients with PD in the BW task. In addition, a larger sample size would make the results robust.

Incorporating a larger sample size, such as different subtypes (tremor dominant and postural Instability/gait difficulty groups), and different stages (H-Y 1, 2, 3, 4, or even prodromal PD) may be more helpful in elaborating the gait characteristics of patients with PD in the BW task. In addition, a larger sample size would make the results robust.

While the difference in BW parameters was more pronounced than in FW parameters between mild and moderate-advanced stages, we observed negative associations between total MDS-UPDRS III scores and FW step velocity, step length, stride length, and stride velocity. However, we did not find any association between clinical variables and BW parameters.

Following the one in ten rule [17, 32], only a maximum of two variables were included in the multivariate regression analysis, which is a limitation. While this guideline helps ensuring stability and reliability of the model estimates and limits overfitting, we were not able to include potential confounding factors such as age or sex. Not including such factors limits the generalizability of our findings as it could bias the estimated effects, and confounds/masks the genuine relationship between gait parameters and clinical PD scores.

Furthermore, the sex distribution was skewed, with 70% of participants being male. Consequently, our results may not be readily generalized to females, and we did not perform a sex-based comparison, which could be particularly interesting in the context of BW, given the observed sex differences in gait speed, stride, and step length [23]. Another limitation is the absence of a comparison between the left and right limbs. Given the motor asymmetries in PD patients, assessing gait parameters for each leg could provide valuable insights. Moreover, we did not perform a cognitive evaluation of participants that could have helped elucidating further the impact of different disease manifestations on gait types. Finally, all participants were evaluated in the ON-medication state, and it is recognized that medication may impact gait parameters. Consequently, an assessment in the OFF-medication state could offer additional insights into the effects of medication on gait. Despite these limitations, our study contributes valuable information regarding gait parameter disparities across different PD stages in BW.

## Conclusions

In conclusion, our study revealed distinct variations in gait parameters between PD patients classified within H&Y stages 1–2 and those in stages 3–4. Patients with H&Y stages 3–4 exhibited more pronounced impairments in both FW and BW compared to those in stages 1–2, with BW presenting more conspicuous deficits. Specifically, step length, stride length, and walk ratio deteriorated as PD stage increases. We found significant correlations between FW parameters and clinical scores, differently from BW. Although these correlations were generally low to moderate, it is evident that further research exploring gait parameters across each PD disease stage is warranted to enhance our understanding of the disease progression and inform clinical interventions. In addition, we did not directly compare BW to FW in this specific study, existing literature consistently reports a decline in step length, stride length, and velocity in FW as PD progresses. Our findings in BW suggest a different pattern of adaptation or impairment, highlighting that the impact of PD on gait is not uniform across different walking directions. Future research directly comparing BW and FW within the same PD population and exploring the underlying neural mechanisms driving these differences would provide valuable insights into the complexities of gait control in in patients with early or even prodromal PD which may improve the early diagnosis of PD which is a clinical challenge.

## Supplementary Information

Supplementary Material 1.

Supplementary Material 2.

Supplementary Material 3.

## Data Availability

The datasets generated during the current study are not publicly available due to GDPR restrictions on patient data but anonymized data are available from the corresponding author on reasonable request.

## References

[1] Aarsland D, Batzu L, Halliday GM, Geurtsen GJ, Ballard C, Chaudhuri R, Weintraub D. Parkinson disease-associated cognitive impairment. Nat Rev Dis Primers. 2021;7(1):1–21. 10.1038/s41572-021-00280-3. 34210995 10.1038/s41572-021-00280-3

[2] Bayle N, Patel AS, Crisan D, Guo LJ, Hutin E, Weisz DJ, Moore ST, Gracies JM. Contribution of step length to increase walking and turning speed as a marker of Parkinson’s disease progression. PLoS One. 2016;11(4):1–13. 10.1371/journal.pone.0152469. 10.1371/journal.pone.0152469 PMC4844147 27111531

[3] Bryant MS, Rintala DH, Hou JG, Lai EC, Protas EJ. Effects of levodopa on forward and backward gait patterns in persons with Parkinson’s disease. Neurorehabil. 2011;29(3):247–52. 10.3233/NRE-2011-0700. 10.3233/NRE-2011-0700 PMC3391536 22142758

[4] Bryant MS, Rintala DH, Hou JG, Collins RL, Protas EJ. Gait variability in Parkinson’s disease: levodopa and walking direction. Acta Neurol Scand. 2016;134(1):83–6. 10.1111/ane.12505. 26399376 10.1111/ane.12505 PMC4805494

[5] Combs SA, Diehl MD, Filip J, Long E. Short-distance walking speed tests in people with Parkinson disease: reliability, responsiveness, and validity. Gait Posture. 2014;39(2):784–8. 10.1016/j.gaitpost.2013.10.019. 24246801 10.1016/j.gaitpost.2013.10.019

[6] Correno MB, Hansen C, Chardon M, Milane T, Bianchini E, Vuillerme N. Association between backward walking and cognition in Parkinson disease: a systematic review. Int J Environ Res Public Health. 2022. 10.3390/ijerph191912810. 36232110 10.3390/ijerph191912810 PMC9566137

[7] Creaby MW, Cole MH. Gait characteristics and falls in Parkinson’s disease: a systematic review and meta-analysis. Parkinsonism Relat Disord. 2018;57:1–8. 10.1016/j.parkreldis.2018.07.008. 30041848 10.1016/j.parkreldis.2018.07.008

[8] Curtze C, Nutt JG, Carlson-Kuhta P, Mancini M, Horak FB. Levodopa is a double-edged sword for balance and gait in people with Parkinson’s disease. Mov Disord. 2015;30(10):1361–70. 10.1002/mds.26269. 26095928 10.1002/mds.26269 PMC4755510

[9] Devos D, Defebvre L, Bordet R. Dopaminergic and non-dopaminergic Pharmacological hypotheses for gait disorders in parkinson’s disease. Fundamental Clin Pharmacol. 2010;24(4):407–21. 10.1111/j.1472-8206.2009.00798.x. 10.1111/j.1472-8206.2009.00798.x 20163480

[10] Di Biase L, Di Santo A, Caminiti ML, De Liso A, Shah SA, Ricci L, Di Lazzaro V. Gait analysis in parkinson’s disease: an overview of the most accurate markers for diagnosis and symptoms monitoring. Sens (Switzerland). 2020;20(12):1. 10.3390/s20123529. 10.3390/s20123529 PMC7349580 32580330

[11] Fritz NE, Worstell AM, Kloos AD, Siles AB, White SE, Kegelmeyer DA. Backward walking measures are sensitive to age-related changes in mobility and balance. Gait Posture. 2013;37(4):593–7. 10.1016/j.gaitpost.2012.09.022. 23122938 10.1016/j.gaitpost.2012.09.022

[12] Gilmore G, Gouelle A, Adamson MB, Pieterman M, Jog M. Forward and backward walking in Parkinson disease: a factor analysis. Gait Posture. 2019;74(August):14–9. 10.1016/j.gaitpost.2019.08.005. 31437733 10.1016/j.gaitpost.2019.08.005

[13] Hackney ME, Earhart GM. Backward walking in Parkinson disease. Mov Disord. 2009;24(2):218–23. 10.1002/mds.22330.Backward. 18951535 10.1002/mds.22330 PMC2945224

[14] Hackney ME, Earhart GM. The effects of a secondary task on forward and backward walking in Parkinson’s disease. Neurorehabil Neural Repair. 2010;24(1):97–106. 10.1177/1545968309341061. 19675121 10.1177/1545968309341061 PMC2888719

[15] Hatanaka N, Sato K, Hishikawa N, Takemoto M, Ohta Y, Yamashita T, Abe K. Comparative gait analysis in progressive supranuclear palsy and parkinson’s disease. Eur Neurol. 2016;75(5–6):282–9. 10.1159/000445111. 27288001 10.1159/000445111

[16] Hoehn MM, Yahr MD. Parkinsonism: onset, progression, and mortality. Neurology. 1967;17(5):427–42. 6067254 10.1212/wnl.17.5.427

[17] Jenkins DG, Quintana-Ascencio PF. A solution to minimum sample size for regressions. PLoS One. 2020;15(2):e0229345. 10.1371/journal.pone.0229345. 32084211 10.1371/journal.pone.0229345 PMC7034864

[18] Katzel LI, Ivey FM, Sorkin JD, MacKo RF, Smith B, Shulman LM. Impaired economy of gait and decreased six-minute walk distance in Parkinson’s disease. Parkinson’s Disease. 2012. 10.1155/2012/241754. 21922051 10.1155/2012/241754 PMC3171762

[19] Kirkwood RN, de Souza Moreira B, Vallone MLDC, Mingoti SA, Dias RC, Sampaio RF. Step length appears to be a strong discriminant gait parameter for elderly females highly concerned about falls: a cross-sectional observational study. Physiotherapy. 2011;97(2):126–31. 10.1016/j.physio.2010.08.007. 21497246 10.1016/j.physio.2010.08.007

[20] Konczak J, Corcos DM, Horak F, Poizner H, Shapiro M, Tuite P, Volkmann J, Maschke M. Proprioception and motor control in parkinson’s disease. J Motor Behav. 2009;41(6):543–52. 10.3200/35-09-002 19592360

[21] Kwon KY, Park S, Lee HM, Park YM, Kim JJ, Kim JJ, Koh SB, Article O. Fear of falling in patients with de Novo Parkinson ’ s disease. J Clin Neurol (Seoul Korea). 2019;15(4):473–9. 10.3988/jcn.2019.15.4.473 PMC6785475 31591835

[22] Lai YR, Lien CY, Huang CC, Lin WC, Chen YS, Yu CC, Cheng BC, Kung C, Te, Kung CF, Chiang YF, Hung YT, Chang HW, Lu CH. Clinical disease severity mediates the relationship between Stride length and speed and the risk of falling in parkinson’s disease. J Personalized Med. 2022;12(2). 10.3390/jpm12020192. 10.3390/jpm12020192 PMC8875632 35207680

[23] Laufer Y. Age- and gender‐related changes in the temporal‐spatial characteristics of forwards and backwards gaits. Physiotherapy Res Int. 2003;8(3):131–42. 10.1002/pri.281. 10.1002/pri.281 14533369

[24] Lee M, Kim J, Son J, Kim Y. Kinematic and kinetic analysis during forward and backward walking. Gait Posture. 2013;38(4):674–8. 10.1016/j.gaitpost.2013.02.014. 23541766 10.1016/j.gaitpost.2013.02.014

[25] Martínez-Martín P, Rodríguez-Blázquez C, Alvarez M, Arakaki T, Arillo VC, Chaná P, Fernández W, Garretto N, Martínez-Castrillo JC, Rodríguez-Violante M, Serrano-Dueñas M, Ballesteros D, Rojo-Abuin JM, Chaudhuri KR, Merello M. Parkinson’s disease severity levels and MDS-Unified parkinson’s disease rating scale. Parkinsonism Relat Disorders. 2015;21(1):50–4. 10.1016/j.parkreldis.2014.10.026. 10.1016/j.parkreldis.2014.10.026 25466406

[26] Milane T, Hansen C, Chardon M, Bianchini E, Vuillerme N. Comparing backward walking performance in Parkinson’s disease with and without freezing of gait—a systematic review. Int J Environ Res Public Health. 2023. 10.3390/ijerph20020953. 36673709 10.3390/ijerph20020953 PMC9859157

[27] Mirelman A, Bonato P, Camicioli R, Ellis TD, Giladi N, Hamilton JL, Hass CJ, Hausdorff JM, Pelosin E, Almeida QJ. Gait impairments in Parkinson’s disease. Lancet Neurol. 2019;18(7):697–708. 10.1016/S1474-4422(19)30044-4. 30975519 10.1016/S1474-4422(19)30044-4

[28] Morio Y, Izawa K, Omori Y, Katata H, Ishiyama D, Koyama S, Yamano Y. The relationship between walking speed and step length in older aged patients. Diseases. 2019;7(1): 17. 10.3390/diseases7010017. 30717332 10.3390/diseases7010017 PMC6473831

[29] Myers PS, Rawson KS, Harrison EC, Horin AP, Sutter EN, McNeely ME, Earhart GM. Cross-sectional analysis of backward, forward, and dual task gait kinematics in people with Parkinson disease with and without freezing of gait. J Appl Biomech. 2020;36(2):85–95. 10.1123/JAB.2019-0253. 32106081 10.1123/jab.2019-0253

[30] Peterson DS, Plotnik M, Hausdorff JM, Earhart GM. Evidence for a relationship between bilateral coordination during complex gait tasks and freezing of gait in parkinson’s disease. Parkinsonism Relat Disorders. 2012;18(9):1022–6. 10.1016/j.parkreldis.2012.05.019. 10.1016/j.parkreldis.2012.05.019 PMC3587964 22717367

[31] Raffegeau TE, Krehbiel LM, Kang N, Thijs FJ, Altmann LJP, Cauraugh JH, Hass CJ. A Meta-Analysis: parkinson’s disease and Dual-Task walking. Parkinsonism Relat Disord. 2019;62:28–35. 10.1016/j.parkreldis.2018.12.012.A. 30594454 10.1016/j.parkreldis.2018.12.012 PMC8487457

[32] Schmidt AF, Finan C. Linear regression and the normality assumption. J Clin Epidemiol. 2018;98:146–51. 10.1016/j.jclinepi.2017.12.006. 29258908 10.1016/j.jclinepi.2017.12.006

[33] Schober P, Schwarte LA. Correlation coefficients: appropriate use and interpretation. Anesth Analg. 2018;126(5):1763–8. 10.1213/ANE.0000000000002864. 29481436 10.1213/ANE.0000000000002864

[34] Sofuwa O, Nieuwboer A, Desloovere K, Willems AM, Chavret F, Jonkers I. Quantitative gait analysis in Parkinson’s disease: comparison with a healthy control group. Arch Phys Med Rehabil. 2005;86(5):1007–13. 10.1016/j.apmr.2004.08.012. 15895349 10.1016/j.apmr.2004.08.012

[35] Son M, Cheon SM, Youm C, Kim Y, Kim JW. (2018). Impacts of freezing of gait on forward and backward gait in Parkinson’s disease. Gait and Posture, 61(November 2017), 320–324. 10.1016/j.gaitpost.2018.01.034 10.1016/j.gaitpost.2018.01.034 29413804

[36] Son M, Cheon SM, Youm C, Kim JW. Turning reveals the characteristics of gait freezing better than walking forward and backward in Parkinson’s disease. Gait Posture. 2022;94(March):131–7. 10.1016/j.gaitpost.2022.03.009. 35306381 10.1016/j.gaitpost.2022.03.009

[37] Sutter EN, Seidler KJ, Duncan RP, Earhart GM, McNeely ME. Low to moderate relationships between gait and postural responses in Parkinson disease. J Rehabil Med. 2017;49(6):505–11. 10.2340/16501977-2238. 28553677 10.2340/16501977-2238 PMC7003247

[38] Toots A, Domellöf ME, Lundin-Olsson L, Gustafson Y, Rosendahl E. Backward relative to forward walking speed and falls in older adults with dementia. Gait Posture. 2022;96:60–6. 10.1016/J.GAITPOST.2022.05.013. 35576668 10.1016/j.gaitpost.2022.05.013

[39] Tseng IJ, Jeng C, Yuan RY. Comparisons of forward and backward gait between poorer and better attention capabilities in early Parkinson’s disease. Gait Posture. 2012;36(3):367–71. 10.1016/j.gaitpost.2012.03.028. 22627144 10.1016/j.gaitpost.2012.03.028

[40] Vargha A, Delaney HD. A critique and improvement of the CL common language effect size statistics of mcgraw and wong. J Educ Behav Stat. 2000;25(2):101–32. 10.3102/10769986025002101.

[41] Vila MH, Pérez R, Mollinedo I, Cancela JM. Analysis of gait for disease stage in patients with Parkinson’s disease. Int J Environ Res Public Health. 2021;18(2):1–10. 10.3390/ijerph18020720. 10.3390/ijerph18020720 PMC7830506 33467634

[42] Wang Y, Witchalls J, Preston E, Wang Z, Zhuang J, Waddington G, Adams R, Han J. The relationship between ankle proprioception and functional mobility in people with Parkinson’s disease: a cross-sectional study. Front Neurol. 2021;11(January):1–7. 10.3389/fneur.2020.603814. 10.3389/fneur.2020.603814 PMC7844086 33519682

[43] Warmerdam E, Romijnders R, Geritz J, Elshehabi M, Maetzler C, Otto JC, Reimer M, Stuerner K, Baron R, Paschen S, Beyer T, Dopcke D, Eiken T, Ortmann H, Peters F, von der Recke F, Riesen M, Rohwedder G, Schaade A, Hansen C. Proposed mobility assessments with simultaneous full-body inertial measurement units and optical motion capture in healthy adults and neurological patients for future validation studies: study protocol. Sensors. 2021;21(17):5833. 10.3390/s21175833. 34502726 10.3390/s21175833 PMC8434336

[44] Warmerdam E, Hansen C, Romijnders R, Hobert MA, Welzel J, Maetzler W. Full-body mobility data to validate inertial measurement unit algorithms in healthy and neurological cohorts. Data. 2022;7(10):136. 10.3390/data7100136.

[45] Welzel J, Wendtland D, Warmerdam E, Romijnders R, Elshehabi M, Geritz J, Berg D, Hansen C, Maetzler W. Step length is a promising progression marker in Parkinson’s disease. Sensors (Basel). 2021;21(7):1–9. 10.3390/s21072292. 10.3390/s21072292 PMC8037757 33805914

[46] Yogev-Seligmann G, Hausdorff JM, Giladi N. The role of executive function and attention in gait. Mov Disord. 2008;23(3):1–28. 10.1002/mds.21720.The. 18058946 10.1002/mds.21720 PMC2535903

