# Supplementary material for: Impact of forward and backward walking on gait parameters across parkinson’s disease stages and severity: a prospective observational study
Source: BMC Neurol. 2025 Sep 9;25:379. doi: 10.1186/s12883-025-04321-2 (PMC12418675; doi:10.1186/s12883-025-04321-2)
Supplement: Supplementary file 1 — Supplementary Material 1. [file 12883_2025_4321_MOESM1_ESM.docx]

**Table S1.** Gait parameters assessed in forward and backward walking

| **Assessed parameters** | **Definition** |
| --- | --- |
| Step frequency [Hz] | The number of steps taken per unit of time during walking. |
| Step velocity [m/s] | The speed at which participants walked, calculated as the distance covered per unit of time during each step of forward walking. |
| Step length [m] | The distance between the heel of one foot and the heel of the other foot during each step of both forward and backward walking. |
| Step duration [s] | The duration of a step, measured as the time between two consecutive foot contacts. |
| Stride velocity [m/s] | The speed at which participants walked, calculated as the distance covered per unit of time during each stride of walking. |
| Stride length [m] | The distance between the heel of one foot and the heel of the same foot in the subsequent step during walking. |
| Stride duration [s] | The duration of the step, measured as the time between two consecutive foot contacts of the same foot. |
| Swing duration [s] | The duration of the swing phase, measured as the time between foot-off and foot contact. |
| Stance duration [s] | The duration of the stance phase, measured as the time between foot contact and foot-off. |
| Walk ratio | The walk ratio is calculated by dividing step length by cadence. |
